# Prevention of polydimethylsiloxane microsphere migration using a mussel-inspired polydopamine coating for potential application in injection therapy

**DOI:** 10.1371/journal.pone.0186877

**Published:** 2017-11-02

**Authors:** Eun-Jae Chung, Dae-Ryong Jun, Dong-Wook Kim, Mi-Jung Han, Tack-Kyun Kwon, Sung-Wook Choi, Seong Keun Kwon

**Affiliations:** 1 Department of Otorhinolaryngology, College of Medicine, Seoul National University Hospital, Seoul, Korea; 2 Department of Biotechnology, The Catholic University of Korea, Gyeonggi-do, Korea; Brandeis University, UNITED STATES

## Abstract

The use of injectable bulking agents is a feasible alternative procedure for conventional surgical therapy. In this study, poly(dimethylsiloxane) (PDMS) microspheres coated with polydopamine (PDA) were developed as a potential injection agent to prevent migration in vocal fold. Uniform PDMS microspheres are fabricated using a simple fluidic device and then coated with PDA. Cell attachment test reveals that the PDA-coated PDMS (PDA-PDMS) substrate favors cell adhesion and attachment. The injected PDA-PDMS microspheres persist without migration on reconstructed axial CT images, whereas, pristine PDMS locally migrates over a period of 12 weeks. The gross appearance of the implants retrieved at 4, 8, 12 and 34 weeks indicates that the PDA-PDMS group maintained their original position without significant migration until 34 weeks after injection. By contrast, there is diffuse local migration of the pristine PDMS group from 4 weeks after injection. The PDA-coated PDMS microspheres can potentially be used as easily injectable, non-absorbable filler without migration.

## Introduction

Soft-tissue augmentation with injectable bulking agents is a feasible alternative procedure for conventional surgical therapy for various diseases. In addition to urinary applications (urinary incontinence and vesicoureteral reflux), injection therapy could be applied to aesthetic applications such as vocal fold paralysis or other treatments that require tissue augmentation. Injection materials for these purposes include collagen, hyaluronic acid, calcium hydroxyapatite (CaHA), polymethylmethacrylate, polytetrafluoroethylene (PTFE), and silicone.[1–5] These injection procedures offer many advantages over conventional surgical options, including reduced morbidity, lower medical costs, a short time operation, and easier operation.[6–8]

An ideal injection substance should be easily injectable, volume-stable, nonantigenic, and non-migratory. Silicone has been clinically used in the past several decades in both the fluid and resin forms. One of the most common silicones useful for biomedical applications is poly(dimethylsiloxane) (PDMS).[9] PDMS-based materials are used for diverse applications including cosmetic dermal fillers, bulking agents, and small joint implants.[10–12] However, it was reported that PDMS and silicone micro-implant particles could migrate to local and distant sites [13,14]. The migration of the injected materials is the crucial drawback for the clinical applications.[9,13,14] Generally, particles with a size less than 70 μm tend to easily migrate. In addition, particle migration can occur through phagocytes that can take up particles up to 20 μm in size in vitro.[15] As the particle size increases to overcome this critical problem, the injection process would become more difficult. Mittleman et al. reported that pulmonary polytetrafluoroethylene granuloma was identified at autopsy 4 years after polytetrafluoroethylene injection for urinary incontinence.[16] Polytetrafluoroethylene particle migration and granuloma formation in the pelvic lymph nodes, lungs, brain, kidneys, choroid plexus and spleen has been reported in animal experiments.[13] DeHeer et al. injected silicone microimplants into female dogs to evaluate particle migration.[11] In all dogs, the injected silicone microimplants were found in the lungs, lymph nodes, kidney and brain.

In an effort to improve the biocompability and cell attachment, there have been several reports on simple surface modification techniques inspired by dopamine that can be readily polymerized on the PDMS surface.[17] The process is known as mussel-inspired coating because the key functional group responsible for the adhesive mechanism of marine mussels is dopamine.[18] The biomimetic oligopeptide modification using polydopamine (PDA) was found to provide a favorable environment for cell adhesion and proliferation due to its hydrophilic property.[19]

In this study, PDMS was chosen as an injection material due to its high biocompatibility, bioinertness, and elasticity.[20–21] However, its extreme hydrophobic property limited their diverse biomedical applications. To overcome its inherent hydrophobicity, we developed PDA-coated PDMS microspheres as a potential bulking agent for soft-tissue augmentation to prevent the migration of injected PDMS microspheres.

## Experimental section

### Materials

The PDMS precursor (Sylgard 184; Sewang Hitech, Korea) and poly(vinyl alcohol) (PVA; M_w_≈13,000–23,000; 98% hydrolyzed; Sigma–Aldrich, USA) were materials that were used for the continuous and discontinuous phases, respectively. The Sylgard mixture was prepared immediately before use according to the manufacturer’s instructions. A solvent medium for Sylgard 184 was dichloromethane (Junsei, Japan). A glass capillary (Ace Glass, USA), syringe needle (BD Medical, USA), and Tygon^®^ tube (Saint-GobainCorp., USA) were used as a simple fluidic device for fabrication. We used dopamine hydrochloride (Sigma–Aldrich, USA) for the surface modification of PDMS microspheres.

### Fabrication of PDA-coated PDMS microspheres

A simple fluidic device was fabricated by assembling a syringe needle (30G), a glass microcapillary (0.5 mm i.d. × 0.9 mm o.d.), and a Tygon^®^ tube (1/32 in i.d. × 3/32 in o.d.), as previously reported.[17] A PDMS precursor solution in DCM (5 wt%) and an aqueous PVA solution (3 wt%) were used as the discontinuous and continuous phases in the fluidic device. Both the discontinuous and continuous phases were introduced into the fluidic device using two syringe pumps (NE-1000; New Era Pump Systems Inc., USA) at the flow rates of 0.05 and 0.5 mL min^-1^, respectively. The resultant PDMS droplets were collected in a tall beaker (1 L) containing the aqueous PVA solution (collection phase). The temperature of the collection phase was kept at 40°C for 3 h to evaporate DCM and then increased to 80°C 2 h to crosslink PDMS. The crosslinked PDMS microspheres were washed with water three times to remove PVA. ImageJ software (National Institutes of Health, USA) was used to evaluate the average size and standard deviation of the PDMS microspheres (n = 300) from optical microscopy images. For PDA-coated PDMS microspheres, the PDMS microspheres (0.5 g) were stirred in a solution (10 mL) of dopamine hydrochloride (0.5 wt%) and Tris–HCl (1 M, 10 mL, pH 8.5) for 3 days using an orbital shaker.[17] Scanning electron microscopy (SEM; S-4800; Hitachi, Tokyo, Japan) was used to observe the surface morphology of the PDMS microspheres.

### Cell adhesion on PDMS and PDA-PDMS substrate

The PDMS precursor was poured into a Petri dish and cured in an oven at 50°C for 12 h. The cured PDMS substrate was cut into 2.5 cm × 2.5 cm; then, half of the PDMS substrate was immersed in a solution of dopamine hydrochloride (0.5 wt%) and Tris–HCl (1 M, 10 mL, pH 8.5) for 3 days. After sterilization with ethanol (70%) and washing with water, the PDMS-based substrate was placed in a Petri dish containing culture media. Mouse NIH-3T3 embryonic fibroblasts (10^5^ cells mL^-1^, ATCC) were seeded on the PDMS-based substrate and cultured in an incubator. After 3 days, the PDMS-based substrate was gently washed three times with water and was then stained with DAPI and rhodamine phalloidin for visualization. The fibroblasts on the PDMS-based substrate were imaged using fluorescence microscopy (FM; Axio Imager D2; ZEISS, Germany). After fixation with glutaraldehyde and freeze drying, the cells were imaged with scanning electron microscopy (SEM; S-4800; Hitachi, Tokyo, Japan).

### Nude mouse subcutaneous injection

To determine whether the PDA-coated PDMS microspheres can prevent the migration of injected PDMS microsphere in vivo, microspheres were injected into the subcutaneous dorsum of mice. Ten 6-week-old female athymic nude mice (BALB/c-nu/nu; Orient Bio Inc., Korea), weighing 30 to 35 g, were selected to evaluate the volume change of injected pristine and PDA-coated PDMS. The injections were subcutaneously applied into the back of the mice; the right sites for the PDA-coated PDMS and left sites for the pristine PDMS. At 4, 8, 12 and 34 weeks after injection, the lumps that formed under the skin from the injection were widely excised to include the skin beyond the swelling points above the panniculus carnosus layer. After the specimens were dissected, H&E staining was performed to confirm the presence of PDA-coated PDMS and pristine PDMS bioimplants.

### Evaluation of reconstructed images on the microCT system

We used a micro-computed tomography (μCT) scanner (Polaris-G90; NanoFocusRay, Jeonbuk, Korea) to identify the injected material in the nude mouse back 0, 1, 2, 4, and 12 weeks after surgery. The 600 μCT images were acquired (65 kV, 120 μA, 500 ms) and stored in the Digital Imaging and Communication in Medicine (DICOM) format with a resolution of 1,024 × 1,024 × 1,024 pixels. The 3D images of each mouse’s back were reconstructed using specialized software (Lucion; Infinitt Healthcare, Seoul, Korea).

To quantitatively evaluate the migration, aspect ratio of the injected area for PDMS and PDA-PDMS microspheres was measured from the microCT images using ImageJ software (n = 3). The aspect ratio was defined by dividing the longitudinal maximum length of the injected area by the width at the center of the injected area. The aspect ratio was normalized to that at 0 week. Experimental results were presented as means ± standard deviation (SD) for each scaffold. Statistical significance (p<0.05) was determined by analysis of variance (ANOVA).

### Histology of the subcutaneously implanted material

The mice were sacrificed in a humane way at 4, 8, 12 and 34 weeks after injection. The animal’s back specimens were removed and fixed with 10% formaldehyde for 24 h, and the tissue blocks were cut into a thickness of 5 mm using a microtome (Leica RM 2235, Leica Biosystems, Germany). The sections were stained with hematoxylin and eosin (H&E), Alcian Blue staining, and Masson’s trichrome to evaluate the remaining injection materials and to determine the change in the soft-tissue component.

### Animal model of glottal insufficiency

To prepare the unilateral vocal fold palsy animal model, male New Zealand white rabbits, weighing 2.6 to 3.0 kg (Koatech Laboratory Animal Company, Republic of Korea), were used. Rabbits were anesthetized with a combination of zoletil (50 mg/kg) and xylazine (4.5 mg/kg), which were administered intramuscularly. A vertical skin incision was made at the midline of the neck, and subcutaneous fat and the strap muscles were separated until the thyroid gland and tracheal cartilage were exposed. The left recurrent laryngeal nerve was identified at the tracheoesophageal groove, and the length of 2 cm of the nerve was cut. The subcutaneous tissue and skin were then replaced and sutured.

### Vocal fold injection of PDMS microspheres and calcium hydroxylapatite (CaHA)

CaHA is the only approved biocompatible material for vocal fold augmentation by the United States Food and Drug Administration Center for Devices and Radiological Health.[22] We compared the volume change after CaHA and PDA-coated PDMS injection into the vocal fold to verify the lower migration of the injected PDMS microspheres.

At 1 week after recurrent laryngeal nerve sectioning, the rabbits were anesthetized in the same way. All rabbits were divided into two groups, CaHA (n = 5) and PDA coated PDMS (n = 5) groups, using a randomized block design. Each injection was made through a syringe with a 25-G spinal needle under the guidance of a 4.0-mm 30° rigid endoscope (Richards, Knittlingen, Germany). The needle of the syringe was inserted and directed just anterolateral to the vocal process so that the vocal process could medially rotate. There has been no definite back-flow of materials. Postoperatively, each animal was observed for approximately 2 h before it was returned to its cage, where both water and feed were available. The rabbits were given 20 mg/kg kanamycin as prophylaxis for the next 3 days. Clinical signs such as weight loss, cough, sputum production, wheezing, or dyspnea were monitored daily.

### Endoscopic evaluation

A laryngeal endoscopic evaluation was performed using a 4.0-mm, 30-degree rigid endoscope. Digital images of each animal’s vocal fold were taken with a camera (E4500; Nikon, Tokyo, Japan) attached on a rigid endoscope at 0, 1, 2, 4, 8, 12, and 34 weeks after the operation with the same protocol.

### Histology of the larynx and microCT evaluation

At four (n = 4) and 34 (n = 6) weeks after injection, the animals were euthanized by CO_2_ asphyxia under general anesthesia, and the larynges were removed by total laryngectomy. The animals that underwent the total laryngectomy were immediately sacrificed by an overdose of CO_2_ gas. The laryngeal specimens were fixed for 24 hours in 10% formalin, embedded in paraffin, and sectioned serially in the axial plane from the false vocal fold to the subglottis. Sections were deparaffinized and dehydrated in a graded series of ethanols. Histological sections were stained with standard hematoxylin and eosin (H&E), Alcian Blue staining, and Masson’s trichrome to evaluate the remaining injection materials and to identify the immune-inflammatory tissue reaction in the vocal fold (epithelium, lamina propria, and muscular layer). A micro-computed tomography (μCT) scanner (Polaris-G90; NanoFocusRay, Jeonbuk, Korea) was used to identify the injected material in the vocal fold 4 and 34 weeks after the operation with the same protocol.

### Ethics statement

This study was carried out in strict accordance with the guidelines of the Animal Research Committee, Seoul National University Hospital. All protocols and experimental design parameters were reviewed and approved by the Institutional Animal Care and Use Committee of the Seoul National University Hospital (approval number: 14-0123-S1A1).

## Results and discussion

### PDA-PDMS microspheres

**Fig 1:**
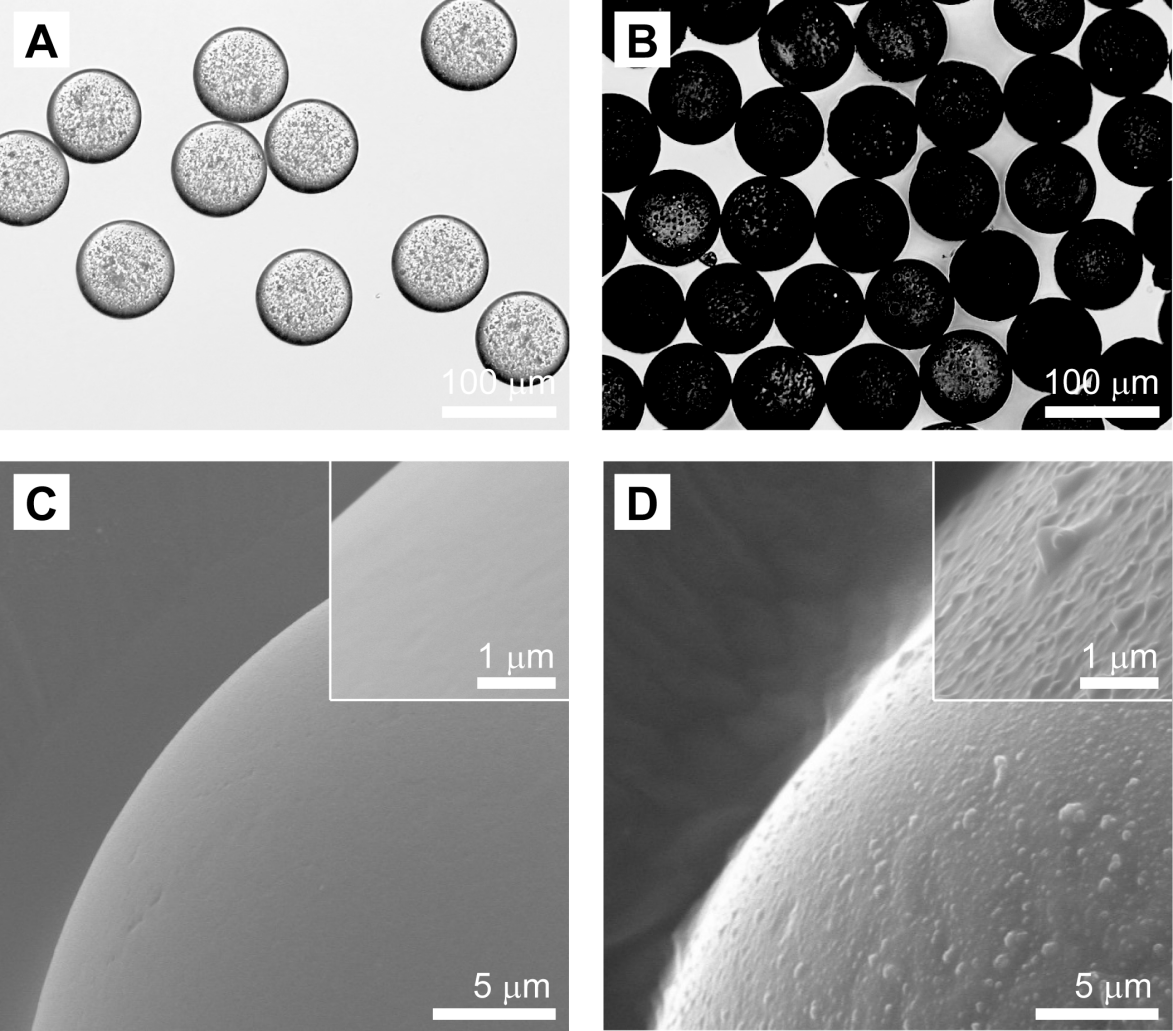
Spherical shape and surface morphology of the PDMS and PDA-PDMS microspheres. Optical microscopy (A, B) and SEM images (C, D) of PDMS (A, C) and PDA-PDMS microspheres(B, D). The insets are magnified SEM images of the surface of the PDMS and PDA-PDMS microspheres.

Resultant PDMS and PDA-PDMS microspheres of 79.33 μm ± 2.23 in size and with a 2.81% coefficient of variation (CV) (Fig 1). The low CV value less than 5% suggests the highly uniform size distribution. Unlike PDMS microspheres, the PDA-PDMS microspheres had a dark surface due to the PDA coating. In addition, PDA-PDMS microspheres had a rough surface morphology, while PDMS microspheres had a smooth surface, as shown in Fig 1C and 1D.

### Cell culture on PDMS and PDA-coated PDMS substrates

It is difficult to achieve the cell attachment on the PDMS surface due to its hydrophobic properties.[23–25] Recently, there have been reports on the process referred to as mussel-inspired coating.[17] This approach is inspired by the adhesion of mussels to rocks in wet environments, and it has been reported that the adhesive proteins secreted by mussels mainly contain dihydroxyphenylalanine (DOPA) and lysine, which has attracted significant attention in the field of biomaterials. Clues to mussels’ adhesive versatility may lie in the amino acid composition of proteins found near the plaque-substrate interface that are rich in 3,4-dihydroxy-L-phenylalanine (DOPA) and lysine amino acids. In addition to participating in reactions that lead to the bulk solidification of the adhesive, DOPA forms strong covalent and noncovalent interactions with substrates.[17] Therefore, the resulting PDMS microspheres were modified with PDA to improve cell attachments, improving the adhesive interaction between host cell/tissue and microspheres. These uniform PDMS microspheres modified with PDA can be novel materials for injection therapy.

**Fig 2:**
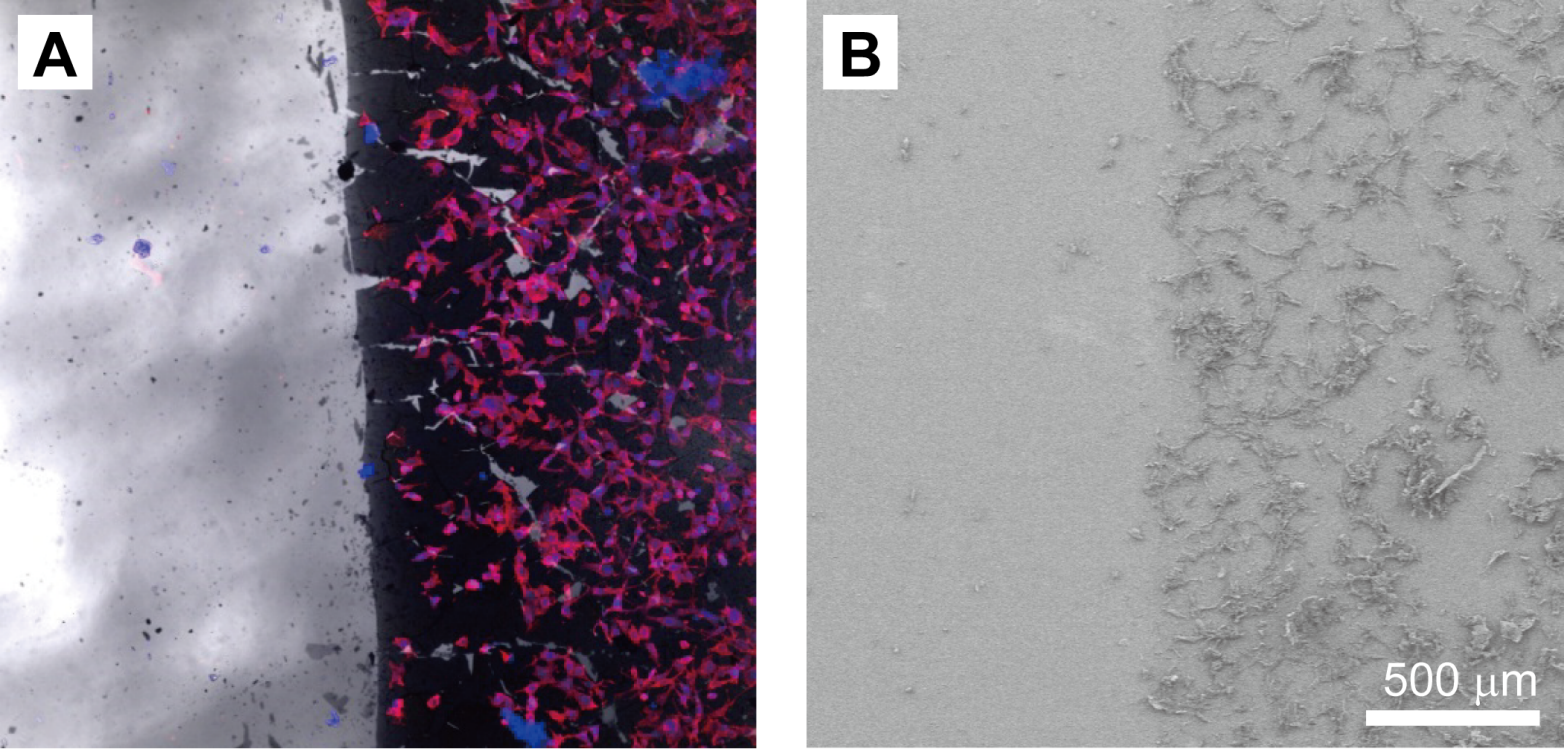
Cell attachment on PDMS and PDA-PDMS substrates. Fluoresce microscopy (A) and SEM images (B) of cells on the PDMS (left half) and PDA-PDMS (right half) substrates.

Fluorescent microscopy and SEM images were collected of the cells cultured on the PDMS-surface side that was coated with PDA (Fig 2). Only a few cells were observed on the PDMS surface (left side in both figures) due to the intrinsic hydrophobicity of PDMS materials. In contrast, many cells were adhered and stretched on the PDA-coated surface (right side in both figures) even after washing with water. This result suggests that the PDA coating facilitated cell adhesion.

### Residual particle analysis and radiological findings from nude mouse subcutaneous injection

**Fig 3:**
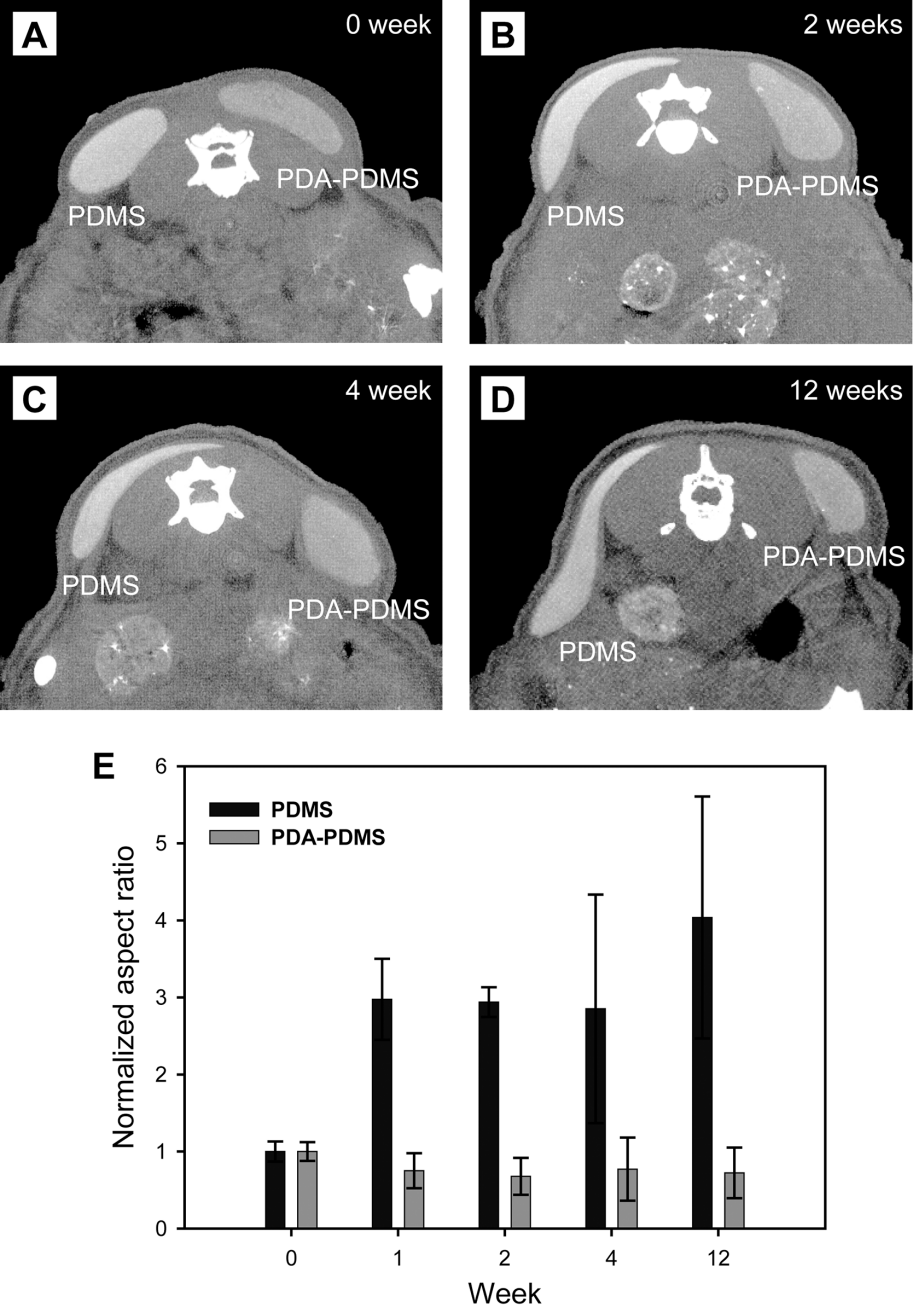
Three-dimensionally reconstructed microCT images. PDA-PDMS microspheres were maintained without migration until 12 weeks after the injection. However, pristine PDMS migrated locally over a period of 12 weeks (A-D). Normalized aspect ratio of the injected area for PDMS and PDA-PDMS microspheres (n = 3) (E).

On the reconstructed axial CT images, PDA-coated PDMS persisted without migration until 12 weeks after injection. However, pristine PDMS migrated locally over a period of 12 weeks **(**Fig 3A–3D**)**. Fig 3E showed the variation of the normalized aspect ratio of the injected area for PDMS and PDA-PDMS microspheres. The normalized aspect ratio of PDMS-injected area was drastically increased to 4.04 at 12 weeks. In contrast, the normalized aspect ratio for the PDA-PDMS microspheres was a little changed over time. This result suggested that the injected area for the PDA-PDMS microspheres maintained its original shape compared to that for the PDMS microspheres, which attributed to the superior cell adhesion and high affinity of the PDA-PDMS microspheres to the host tissue.

### Histological analyses of the subcutaneously implanted material

Although the median diameter of the particles in this study was similar to that previously reported (79. 33 μm ± 2.23), local migration was significantly reduced compared with that of pristine PDMA particles through the PDA-coating process.[11]

**Fig 4:**
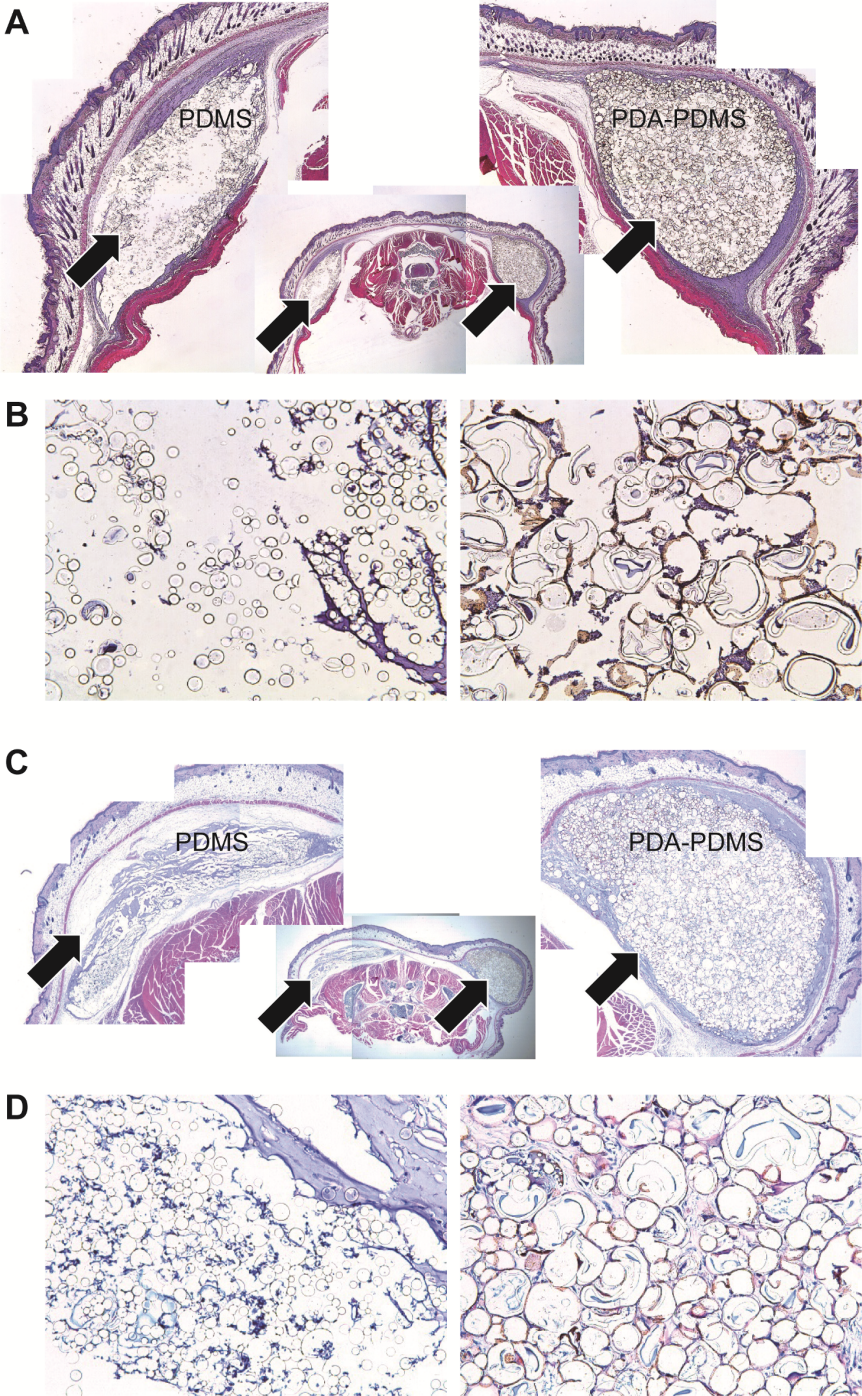
Axial cross-section image of the mouse’s back (H&E stain). Low-power-field views (×12.5) of the implants indicated that the injected PDA-coated PDMS lacked significant migration at 4 weeks (A, right arrow). There was a significant local migration of the pristine PDMS group through the potential fascia space from 4 weeks after injection (A, left arrow). In the PDA-coated PDMS group at 4 weeks, significantly more cells migrated between each microsphere in the high-power-field views (×400) (B). Markedly more residual material was observed in the PDA-coated PDMS group than in the pristine PDMS group at 34 weeks after the injection (C, right arrow). However, most of the injected pristine PDMS was not identified at 34 weeks (C, left arrow) in the low-power-field view (×12.5). Most of the pristine PDMS microspheres hardly adhered to each other. In contrast, the PDA-coated PDMS surface was found to be aggregated until 34 weeks after the injection under the high-power field view (×400) (D).

Low-power-field views (×12.5) of the implants indicated that the injected PDA-coated PDMS lacked significant migration at 4 weeks (Fig 4A, right arrow). In contrast, there was diffuse local migration of the pristine PDMS group through the potential fascia space from 4 weeks after injection (Fig 4A, left arrow). In the PDA-coated PDMS group at 4 weeks, significantly more cells migrated between each microsphere in high-power-field views (×400) (Fig 4B).

We observed markedly more residual material in the PDA-coated PDMS group than in the pristine PDMS group at 34 weeks after injection (Fig 4C, right arrow). However, most injected pristine PDMS was not identified at 34 weeks (Fig 4C, left arrow) in the low power field view (×12.5). There were no significant inflammatory reactions (e.g., hyperemia or granulation formation) in any group until 34 weeks after injection. Host cells from the surrounding tissues migrated to the injected microspheres and formed new hybrid tissue structures in the PDA-coated PDMS group. Aggregation of the pristine PDMS microspheres was observed at 4 weeks due to their inherent hydrophobic property. At 34 weeks, the aggregation became disappeared and more disperse, possibly leading to the migration of the pristine PDMS microspheres. In contrast, the PDA-coated PDMS surface was found to be aggregated with until 34 weeks after the injection in the high-power field view (×400) (Fig 4D).

### Residual particle analysis from paralyzed vocal fold injection and laryngoscopic evaluation

**Fig 5:**
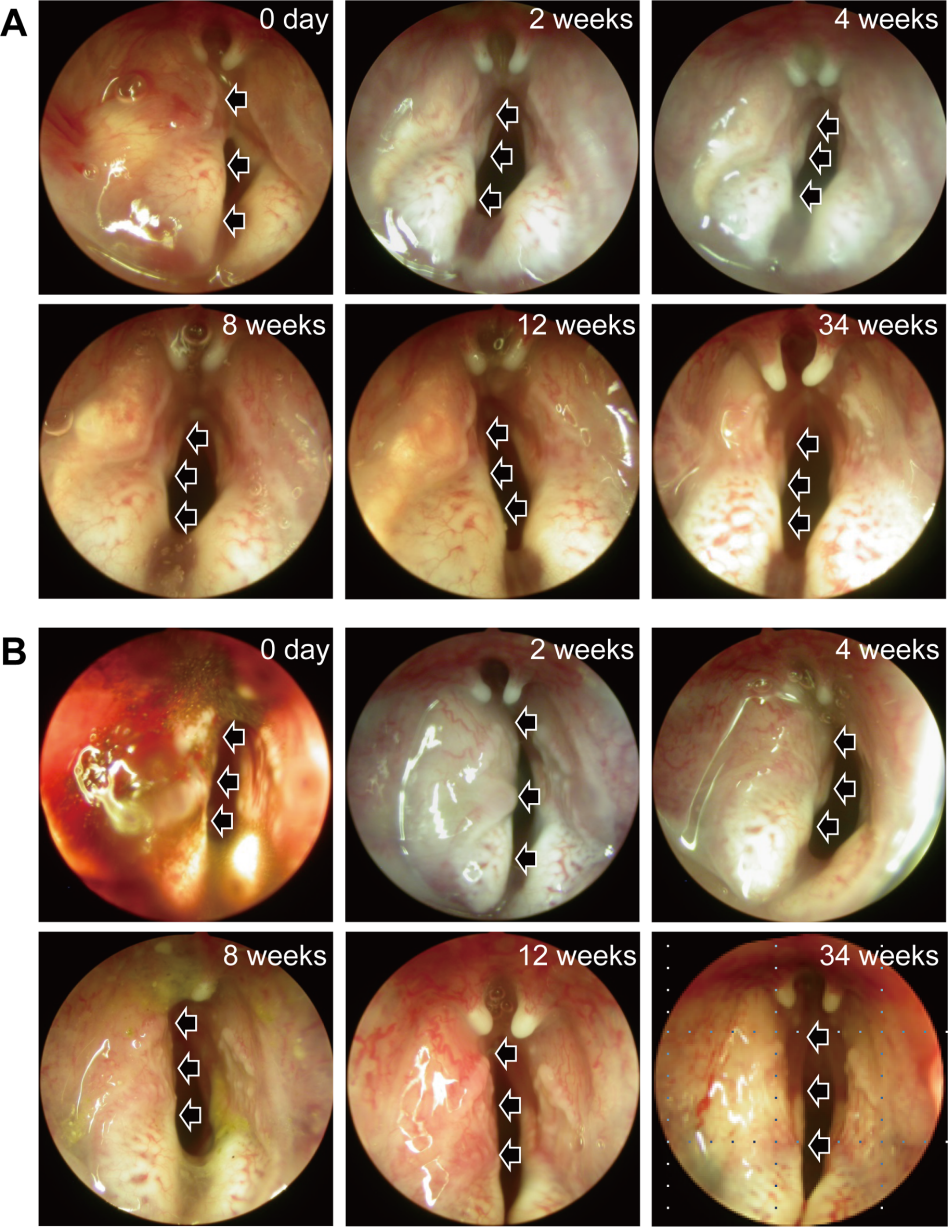
Serial endoscopic analysis of a larynx. CaHA (A) and PDA-coated PDMS (B) microspheres were injected to the larynx. The position of the microspheres were indicated by the black arrows. The images were obtained immediately after the injection; 1 week after the injection; and 2, 4, 8, 12 and 34 weeks after the injection. CaHA leaked out from the puncture site immediately after the injection. Most of the injected PDA-coated PDMS microspheres remained at the injection site. The left vocal folds were augmented significantly greater in the PDA-coated PDMS group than in the CaHA group during the entire follow-up period. In contrast, there was a significant volume reduction of CaHA from the first week after injection.

All animals survived without complications after the injection. Immediately after the injection, all PDA-coated PDMS materials had an augmentation effect, while some CaHA leaked out. The volume of all PDA-coated PDMS material appeared to lack a significant decrease until 34 weeks and the vocal fold maintained in a straight line. In contrast, there was a significant volume reduction of CaHA from the first week after injection. The shape of vocal fold became bowing due to the volume reduction of CaHA. The volume of residual material was significantly larger in the PDA-coated PDMS group than in the CaHA group throughout the follow-up period (Fig 5 and S1 Fig). There was no inflammatory response, such as granulation formation or hyperemia, in any group.

### Histology and microCT of the larynx of the remaining material

**Fig 6:**
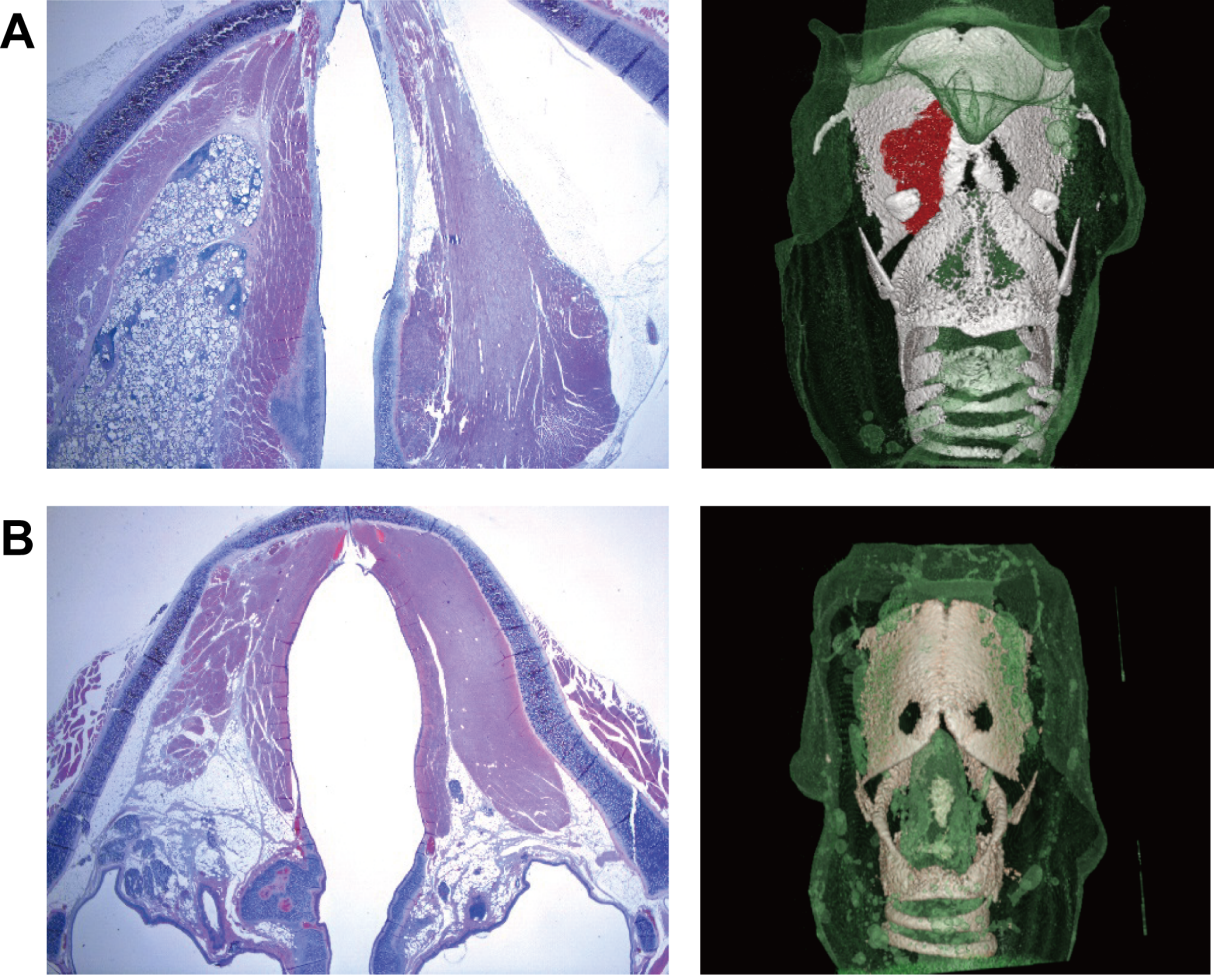
Histology and microCT of the larynx of the remaining material. (A) The injected material in the PDA-coated PDMS group persisted until 34 weeks after the injection without significant migration on histological examination and microCT. (B) Most of the injected CaHA was not identified at 34 weeks.

The injected material in the PDA-coated PDMS group remained until 34 weeks after the injection without significant migration on the histological examination. In contrast, there was an obvious volume reduction of CaHA (Fig 6). On the reconstructed microCT images, PDA-coated PDMS remained at the injection site, but most of the injected CaHA was not identified at 34 weeks.

The volume of injected material was maintained at approximately constant over 34 weeks in the PDA-coated PDMS group by microCT and histological analyses. Although we did not verify the histological analysis of distant migration, our study exhibited no evidence of significant local migration of the PDA-coated PDMS microspheres. These results are promising compared with other injection materials that failed by rapid volume decline, although a long-term study is needed to determine the long-term volume conservation.[26–28]

## Conclusion

We successfully developed mussel-inspired PDA-PDMS microspheres with a uniform size distribution. Unlike pristine PDMS microspheres, CT and gross appearance results revealed that the PDA-PDMS microspheres could inhibit migration in vocal fold, which is attributed to the improved adhesion interactions between the PDA-PDMS microspheres and host cell/tissue. PDA-PDMS microspheres can potentially be used as easily injectable, non-absorbable fillers without migration.

## Supporting information

S1 Fig Serial endoscopic analysis of a larynx injected with CaHA (A, n = 5) and PDA-coated PDMS groups (B, n = 5). (PDF)

S1 File NC3Rs ARRIVE guidelines checklist. (PDF)
