# Supplementary material for: Prevention of polydimethylsiloxane microsphere migration using a mussel-inspired polydopamine coating for potential application in injection therapy
Source: PLoS One. 2017 Nov 2;12(11):e0186877. doi: 10.1371/journal.pone.0186877 (PMC5667927; doi:10.1371/journal.pone.0186877)
Supplement: S1 Fig — (PDF) [file pone.0186877.s001.pdf]

Supporting Information

Prevention of polydimethylsiloxane microsphere migration using a mussel-inspired polydopamine coating for potential application in injection therapy

Eun-Jae Chung[1,¶], Dae-Ryong Jun[2,¶], Dong-Wook Kim[1], Mi-Jung Han[1], Tack-Kyun Kwon[1], Sung-Wook Choi[2,*], and Seong-Keun Kwon[1,*]

[1]Department of Otorhinolaryngology, College of Medicine, Seoul National University Hospital, Seoul, Korea

[2]Department of Biotechnology, The Catholic University of Korea, Gyeonggi-do, Korea

*Corresponding authors

E-mail: otolarynx@snuh.org (S-K Kwon) and choisw@catholic.ac.kr (S-W Choi)

[¶]These authors contributed equally to this work.

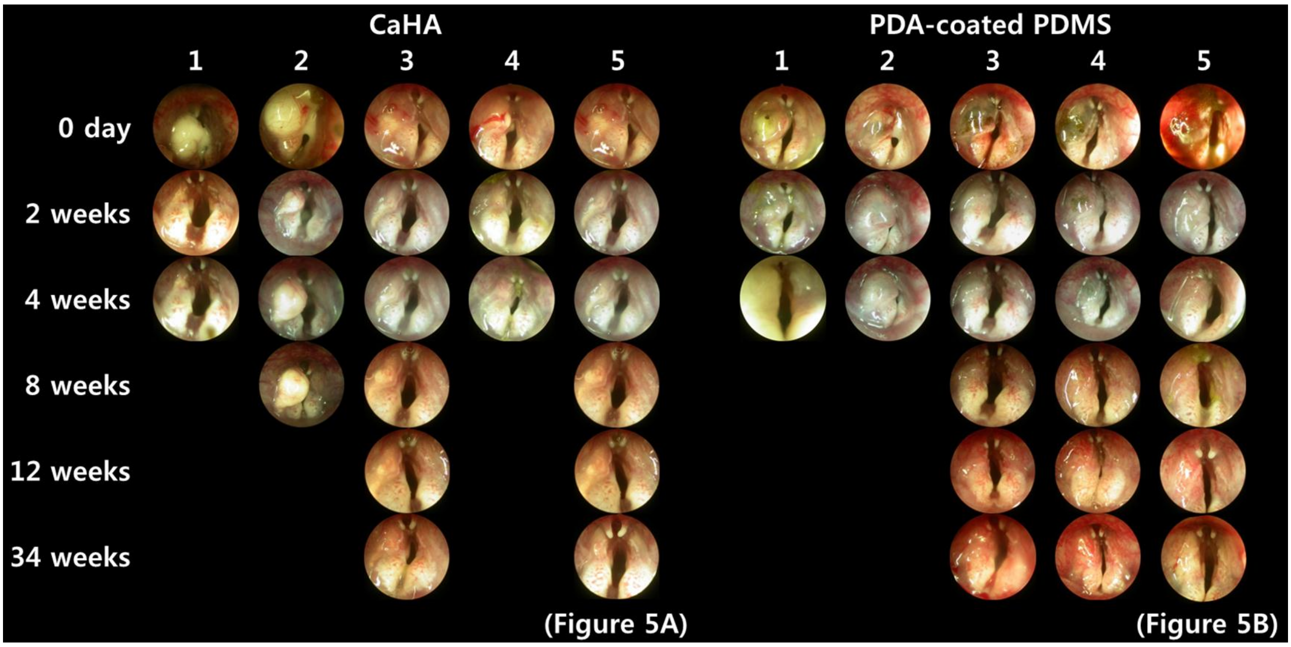

S1 Fig. Serial endoscopic analysis of a larynx injected with CaHA (A, n=5) and PDA-coated PDMS groups (B, n=5).
